# Pericardial Effusion During the Perioperative Period for Left Atrial Appendage Closure

**DOI:** 10.3389/fcvm.2021.678460

**Published:** 2021-08-02

**Authors:** Lifan Yang, Xiaochun Zhang, Qinchun Jin, Dehong Kong, Yuan Zhang, Mingfei Li, Lei Zhang, Shasha Chen, Wenzhi Pan, Daxin Zhou, Junbo Ge

**Affiliations:** ^1^Department of Cardiology, Shanghai Institute of Cardiovascular Disease, Zhongshan Hospital, Fudan University, Shanghai, China; ^2^Department of Echocardiology, Shanghai Institute of Cardiovascular Disease, Zhongshan Hospital, Fudan University, Shanghai, China

**Keywords:** left atrial appendage closure, pericardial effusion, non-valvular atrial fibrillation, pericardial tamponade, paroxysmal AF

## Abstract

**Objectives:** To analyze the predictors of pericardial effusion (PE) during the perioperative period of the left atrial appendage closure procedure in our center.

**Methods:** A total of 624 consecutive patients with non-valvular AF undergoing LAAC from May 2014 to October 2019 were involved in this study. Patients were divided into groups depending on whether they showed no PE, intraoperative PE or postoperative PE. We analyzed the predictors of PE during the perioperative period of the LAAC procedure.

**Results:** (1) Of the 624 patients in our population (age 68.2 ± 9.1 years, 63% male, CHA2DS2-VASc score 3.4 ± 1.6), 30 patients experienced PE in the perioperative period, including 10 intraoperative PE and 20 postoperative PE. (2) A total of 26 (86.6%) patients had mild PE. 4 (13.4%) patients had pericardial tamponade, 2 (6.7%) of which were intraoperative, and the other 2 (6.7%) postoperative. (3) Significant differences were measured in relation to female sex, intraoperative time, combined procedures, changes in sinus rhythm, device retrieval times and duration of hospitalization between 2 groups (no PE occurred, intraoperative PE), *P* values were 0.039, 0.024, 0.004, 0.015, 0.003 and 0.039.

**Conclusions:** Female sex, paroxysmal AF, changing in sinus rhythm, device retrieval times and intraoperative time all had a positive association with PE during the perioperative period.

## Introduction

Atrial fibrillation (AF) is the most common sustained cardiac arrhythmia (1). Of AF's various clinical outcomes, the most severe is thromboembolic stroke. Left atrial appendage closure (LAAC) has emerged as an effective alternative to stroke prevention for patients with AF (2). Currently, percutaneous LAAC is mainly indicated for patients with non-valvular AF and high bleeding risk from anticoagulant therapy (2). These patients may be more vulnerable to procedural complications. Pericardial effusion (PE) is a complication mainly attributed to local interventional trauma, left atrial appendage (LAA) wall thickness and scratching of the inner pericardial membrane (3, 4). The rate of PE has been measured to a range of <1 to 5.2% (1, 5–7). This study aims to explore the predictors of PE for the LAAC procedure and draw lessons from these predictors.

## Methods

A total of 624 consecutive patients with non-valvular AF undergoing LAAC were involved in this study. We analyzed the predictors of PE during the perioperative period of the LAAC procedure. Patients were divided into groups depending on whether they showed no PE, intraoperative PE or postoperative PE. Intraoperative PE was defined as that which occurred during the procedure. Postoperative PE refers to PE that occurred within 24 hours following the procedure. The perioperative period is the period of time spanning from the start of the procedure to 24 hours after the procedure. The procedural predictors of PE were listed as combined procedures, device retrieval, transseptal puncture, tissue rupture and other physical manipulations taking place during the operation.

### Patient Selection

The study was a single-center retrospective analysis of 624 consecutive patients with non-valvular AF undergoing LAAC from May 2014 to October 2019. Non-valvular AF was defined as AF in the absence of rheumatic valvular disease or prosthetic valves. The study was approved by the Biomedical Research Ethics Committee of the Zhongshan Hospital affiliated with Fudan University. The ethics committee waived informed consent for this retrospective analysis.

### Echocardiography

One day prior to the procedure, the patients routinely accepted transthoracic echocardiography (TTE) and transesophageal echocardiography (TEE). The parameters recorded via TTE were: left ventricular ejection fraction, left atrium dimension, LAA ostium dimensions, landing zone diameter (at a depth of 10 mm from the ostium), LAA flow velocities and depth along its contour (0°, 45°, 90°, 135°). If the patients chose general anesthesia, compression ratio and residual flow were recorded via TEE during the LAAC procedure. TTE was performed more than twice to track PE for 24 hours post-procedure. Echocardiography was performed by two independent physicians in our study. The PE volume was assessed quantitatively by M-mode measurement of the end-diastolic echo-free space between the epicardium and the parietal pericardium, from the parasternal short-axis and long-axis views as well as the apical views. PE was classified as mild (<10 mm), moderate (10–20 mm) or severe (>20 mm) according to the guidelines from the European Society of Cardiology (8).

### Implantation Procedures

LAAC means to seal LAA using occlude by percutaneous catheter. It was performed under local anesthesia or general anesthesia and used fluoroscopic guidance, following the description provided in previous academic literature (9). The right, rather than the left, femoral vein is the preferred access site as it allows for favorable orientation of the transseptal sheath. Heparin was administered before or upon transseptal puncture to achieve a target activated clotting time of 250–300s. After making the transseptal puncture, operators observed and measured the LAA using angiography (at a 30° RAO, 20° Cranial projection) and based on these measurements choose the appropriately-sized occlusion device. The sheath was positioned in the LAA such that the distal end of its marker band was located at the intended landing zone. Before device release, the positioning and stability of the device was confirmed via TEE or LAA angiography. The deployment of the device was obtained by pulling back the delivery sheath. After deployment, the device could be partially or fully retrieved if its position or size were deemed inappropriate. Finally, operators measured the device compression ratio, residual leak and released device. Watchman and Lambre occludes were used in the study. Of the 30 PE patients, 16 patients used Watchman occlude and 14 patients had Lambre occlude (*P* = 0.60). There are mainly 2 operators performing LAAC. Their average number of devices implant was about 500 per year.

### Statistical Analysis

Logistic regression analysis was performed by R software. Differences were considered statistically significant at a two-sided *P* value of <0.1. Other statistical analyses were performed using SPSS software, version 22 (SPSS, Inc., Chicago, IL). Continuous were presented as mean value ± standard deviation, and categorical variables were presented as percentages. Either a one-way analysis of variance or a Kruskal-Wallis rank test was used for comparisons between groups. The least significant difference method was used for comparisons within groups. Categorical variables were compared by chi-square test. Differences were considered statistically significant at a two-sided *P* value of <0.05.

## Results

### Baseline Characteristics

**Figure 1:**
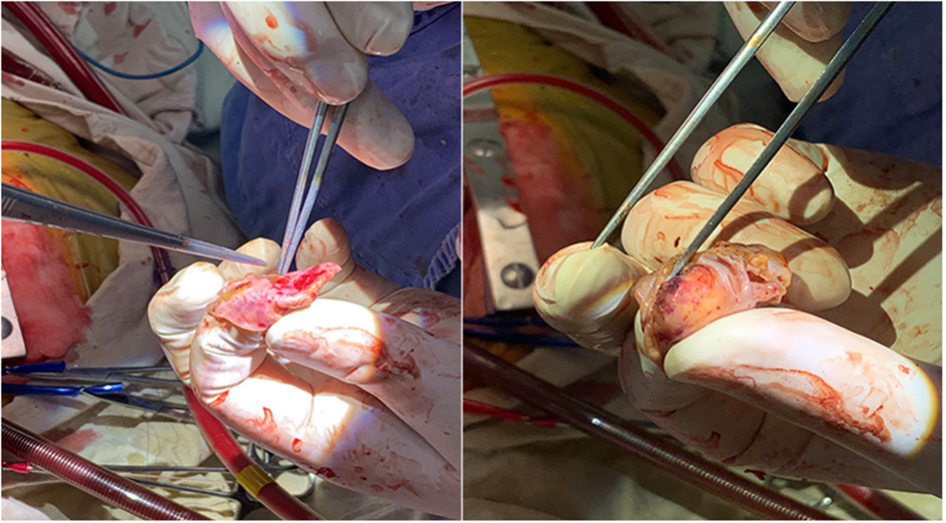
Pericardial tamponade due to tissue rupture. Rupture of left atrial appendage due to massive PE and pericardial tamponade.

This study included 624 patients (age 68.2 ± 9.1 years, 63% male, CHA2DS2-VASc score 3.4 ± 1.6). Among them, 30 patients experienced PE in the perioperative period, including 10 intraoperative PE and 20 postoperative PE. Female patients and hospital stays showed a significant difference in 3 groups (showing no PE, intraoperative PE or postoperative PE), with *P* values of 0.03 and <0.001 (Table 1). Of the 30 PE patients, 26 (86.6%) patients had mild PE. 2 (6.7%) patients experienced pericardial tamponade due to tissue rupture (Figure 1), and another 2 (6.7%) patients had pericardial tamponade because of manipulation during operation (Table 2).

**Table 1:** The baseline data for pericardial effusion in the perioperative period.

	**No. PE(594 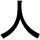 )**	**Intraoperative PE(10 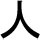 )**	**Postoperative PE(20 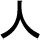 )**	***P* value**
Age (year)	68.2 ± 9.1	70.7 ± 8.2	66.0 ± 9.1	0.313
Female (%)	37	70	50	0.030
Hospital stays (day)	5.9 ± 2.9	10.9 ± 6.6	5.9 ± 3.8	0.000
CHA2DS2-VASC	3.5	3.5	3.2	0.670
HAS-BLED	3.0 ± 1.2	2.4 ± 1.3	2.9 ± 1.0	0.349
Paroxysmal AF (%)	22.3	37.5	14.3	0.261
HBP (%)	63.1	75	78.6	0.106
DM (%)	20	25	26.3	0.321
CAD (%)	16.3	37.5	10.7	0.087
NT-pro BNP (pg/ml)	1,078.0 ± 1,835.5	1,004.0 ± 851.1	1,078.9 ± 892.6	0.992
LAD (mm)	47.1 ± 6.7	49.2 ± 5.7	49.0 ± 6.1	0.220
LAA flow velocities (m/s)	0.4 ± 0.2	0.4 ± 0.2	0.3 ± 0.1	0.660
LVEF (%)	62.9 ± 6.8	61.6 ± 8.6	63.6 ± 4.0	0.718
Mean ostium diameter of LAA (mm)	21.9 ± 7.1	22.0 ± 5.9	24.6 ± 5.7	0.143
Mean depth of LAA (mm)	23.4 ± 4.9	23.5 ± 5.2	25.0 ± 4.6	0.408

*PE, pericardial effusion; HBP, high blood pressure; CAD, coronary atrial disease; NT-pro BNP, N-terminal Brain natriuretic peptide; LAD, left atrial diameter; LAA, left atrial appendage; LVEF, left ventricular ejection fraction*.

**Table 2:** The predictors of pericardial effusion relating to procedural manipulation.

	**Degree of PE**	**Intraoperative PE (10)**	**Postoperative PE (20)**	**PE (30)**
Combined procedures	Mild	3	3	6
Device retrieval	Mild	3	6	9
Trans-septal puncture	Mild	1	0	1
Other manipulation	Mild	1	9	10
	Moderate	1	0	1
	Severe	0	1	1
Tissue rupture	Severe	1	1	2

### Procedural Data

Combined procedures, changes in sinus rhythm and device retrieval times were found to be significantly different in 3 groups (showing no PE, intraoperative PE or postoperative PE) (Table 3). We then performed further comparisons within the groups. Female sex, intraoperative time, combined procedures, changes in sinus rhythm, device retrieval times and hospital stays were significantly different in 2 groups (with no PE or intraoperative PE), which had *P* values of 0.039, 0.024, 0.004, 0.015, 0.003 and 0.039 (Table 4). The logistic regression analysis results showed female, paroxysmal AF, changing in sinus rhythm, device retrieval times and intraoperative time were predictors of PE (Table 5).

**Table 3:** The procedural predictors for pericardial effusion (comparison between groups).

	**No. PE (594 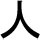 )**	**Intraoperative PE (10 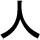 )**	**Postoperative PE (20 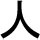 )**	***P* value**
Intraoperative time (min)	63.3 ± 18.1	85.0 ± 24.5	70.7 ± 25.1	0.073
Retrieval (0/1/2 times)	423/138/33	4/3/3	14/5/1	0.021
Compression ratio of TEE	24.0 ± 5.6	26.5 ± 2.6	21.8 ± 6.6	0.392
Compression ratio of DSA	19.6 ± 6.1	17.0 ± 4.6	22.4 ± 4.4	0.138
Double lobes (%)	17.2	12.5	14.3	0.775
ACT	339.7 ± 107.1	268.7 ± 65.7	336.8 ± 103.3	0.283
Residual flow (mm)	1	1	1.6	0.565
Combined procedures	39	3	3	0.007
Changes in sinus rhythm	79	4	6	0.007
Local anesthesia	579	9	19	0.290
Watchman (%)	79.2	75	78.5	0.773

*ACT, activated clotting time*.

**Table 4:** The predictors for pericardial effusion (comparison within groups).

***P* value**	**No. PE & intraoperative PE**	**No. PE & postoperative PE**	**Intraoperative PE & postoperative PE**
Female sex	0.039	0.228	0.278
Hospital stay	0.039	0.161	0.012
Intraoperative time	0.024	0.338	0.115
Combined procedures	0.004	0.142	0.333
Changes in sinus rhythm	0.015	0.033	0.584
Retrieval	0.003	0.980	0.123

**Table 5:** The predictors for pericardial effusion (Logistic regression analysis).

**Baseline predictors**	**z 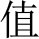 **	***P* value**
Age (year)	1.512	0.181
Female (%)	−2.010	0.044
HBP (%)	1.197	0.231
DM (%)	−0.260	0.795
CAD (%)	−0.629	0.529
Paroxysmal AF (%)	4.589	<0.001
NT-pro BNP (pg/ml)	0.412	0.812
Intraoperative time (min)	1.699	0.089
Retrieval (0/1/2times)	1.441	0.049
Combined procedures	1.365	0.172
Changes in sinus rhythm	1.913	0.056
Residual flow (mm)	0.391	0.696

### Predictors and Treatments of PE

**Figure 2:**
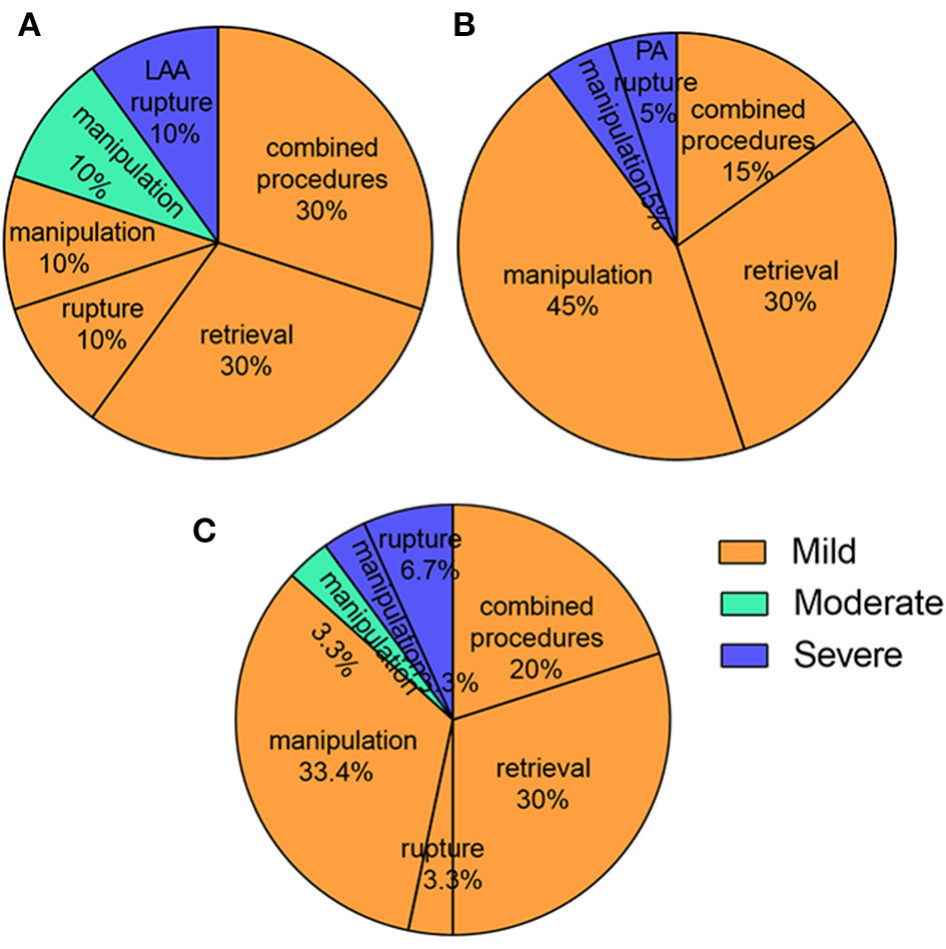
The predictors of PE in the perioperative period. **(A)** Combined procedures and retrievals were the most reasons for intraoperative PE; **(B)** Manipulation and retrievals were the most reasons for postoperative PE; **(C)** Manipulation, combined procedures and retrievals were the most reasons for all PE.

For the 26 (86.6%) mild PE cases, 20% patients were caused by combined procedures and 16.7% patients of them were catheter ablation. 30% were attributed to device retrievals and 33.4% to manipulation during operation (Figure 2), which was of no hemodynamic significance and did not require treatment. 4 (13.4%) patients had serious PE, deemed so because they were of hemodynamic significance and required intervention. Of the 4 patients requiring intervention, 2 were successfully drained percutaneously with a standard subxyphoid or transthoracic puncture approach. The 2 remaining patients underwent surgical intervention attributed to LAA and pulmonary artery perforation, one of which occurred during the procedure, and the other 4 hours afterwards.

After catheter or surgical drainage of the PE, all patients saw good functional recoveries. There was no disability or death observed in relation to PE. For patients requiring either percutaneous or surgical intervention, the mean duration of hospitalization was 17 days.

## Discussion

The primary findings of the study are as follows. (1) Female sex, paroxysmal AF, changing in sinus rhythm, device retrieval times and intraoperative time were found to be predictors of PE during LAAC. (2) Incidence of PE was associated with the level of experience of operators. (3) Intraoperative PE led to comparatively longer hospital stays.

The most common complication for the LAAC procedure is PE, which may be caused during the trans-septal puncture, manipulation of equipment within the LAA, and device deployment and retrievals. The rate of occurrence of PE within 7 days of implantation was measured within a range of <1 to 5.2% (1, 5–7). Our results are superior to previous results that found the rate of PE to be 4.8% and severe PE only 0.32%.

Women who underwent LAAC had a higher risk of PE than men (10), which might result from physiological, electrical, and structural characteristics of the atria (11). For LAAC that involved catheter ablation, female patients were more likely to be older and have higher prevalence of chronic conditions (11). Another reason may be sex hormones. Older women generally have lower levels of estrogen, which has inhibitory effects on pro-inflammatory T cells (12). Thus, it is necessary to take further caution with female patients undergoing LAAC and ensure that sufficient preoperative discussions take place in order to assess their anticipated risk.

PE results in our study indicate the significance of the actual manipulation of the LAA occlusion device itself. In our study, at least one device retrieval was performed in 28.8% of cases with no PE, and 60.0% of those with intraoperative PE (*P* = 0.003). The more experienced the operators were, the less time the operation took. The incidence of PE decreased as the operator's number of previous implantations increased and operator training on LAAC implantation improved (13, 14).

As part of the sinus rhythm, the contraction of the LAA exerts mechanical force on the device that can lead to PE (13). Mechanical contraction can create larger perforations and subsequent higher pericardial blood volume. In our research, changes in sinus rhythm increased the rate of both intraoperative and postoperative PE (*P* = 0.015 and 0.033). Some researchers have suggested that, for high-volume operators, the addition of LAA occlusion to an ablation procedure for AF does not increase the chance of major complications (15). In our study, mild PE occurred in 6 patients who underwent LAAC and combined procedures. No moderate or severe PE was observed in these people.

The occurrence of PE was not associated with a worse clinical outcome, but acute and severe PE were associated with hospital mortality. Severe PE causing hemodynamic compromise requires emergency pericardiocentesis and possibly surgical intervention for cardiac perforation. Moderate PE must be drained and mild PE must be monitored during hospitalization. Compared with no PE and postoperative PE, patients experiencing intraoperative PE, including 8 mild, 1 moderate and 1 severe occurrence of PE, were required to spent longer in hospital (*P* < 0.001). The mean duration of hospitalization was 17 days among moderate and severe PE.

### Strengths and Limitations

This is the first large-scale investigation to study the predictors of PE during the perioperative period of the LAAC procedure. Our study has several limitations. Firstly, the present study was undertaken in a single center and may lead to selection bias. However, our center is one of the three largest heart centers in China, giving us a large sample size consisting of patients from all over the country. Secondly, our cohort of patients with PE was relatively small. Therefore, the distribution of predictors of PE may not accurately reflect the general conditions. Nonetheless, the results can still indicate the possible predictors of PE.

## Conclusion

Female sex, paroxysmal AF, changing in sinus rhythm, device retrieval times and intraoperative time all had a positive association with PE in the perioperative period. In light of this, operators should continue working to gain more experience in order to avoid intraoperative PE and reduce hospital stays.

## Data Availability Statement

The original contributions generated for the study are included in the article/supplementary material, further inquiries can be directed to the corresponding author/s.

## Ethics Statement

The studies involving human participants were reviewed and approved by the Biomedical Research Ethics Committee of the Zhongshan Hospital affiliated with Fudan University. The patients/participants provided their written informed consent to participate in this study.

## Author Contributions

LY, XZ, WP, DZ, and JG contributed to the conceptualization and design. XZ, ML, LZ, SC, and DZ underwent LAAC. QJ, DK, and YZ contributed to the Patient and Public Involvement. LY and XZ completed statistical analysis and led the manuscript preparation. All authors contributed to the writing of the manuscript. All authors read and approved the final manuscript.

## Conflict of Interest

The authors declare that the research was conducted in the absence of any commercial or financial relationships that could be construed as a potential conflict of interest.

## Publisher's Note

All claims expressed in this article are solely those of the authors and do not necessarily represent those of their affiliated organizations, or those of the publisher, the editors and the reviewers. Any product that may be evaluated in this article, or claim that may be made by its manufacturer, is not guaranteed or endorsed by the publisher.
